# Population-level transposable element expression dynamics influence trait evolution in a fungal crop pathogen

**DOI:** 10.1128/mbio.02840-23

**Published:** 2024-02-13

**Authors:** Leen Nanchira Abraham, Ursula Oggenfuss, Daniel Croll

**Affiliations:** 1 Laboratory of Evolutionary Genetics, Institute of Biology, University of Neuchâtel, Neuchâtel, Switzerland; University of Melbourne, Melbourne, Australia

**Keywords:** transposable elements, gene expression, rapid adaptation, plant pathogen, fungi *Zymoseptoria tritici*

## Abstract

The rapid adaptive evolution of microbes is driven by strong selection pressure acting on genetic variation. How adaptive genetic variation is generated within species and how such variation influences phenotypic trait expression is often not well understood though. We focused on the recent activity of transposable elements (TEs) using deep population genomics and transcriptomics analyses of a fungal plant pathogen with a highly active content of TEs in the genome. *Zymoseptoria tritici* causes one of the most damaging diseases on wheat, with recent adaptation to the host and environment being facilitated by TE-associated mutations. We obtained genomic and RNA-sequencing data from 146 isolates collected from a single wheat field. We established a genome-wide map of TE insertion polymorphisms in the population by analyzing recent TE insertions among individuals. We quantified the locus-specific transcription of individual TE copies and found considerable population variation at individual TE loci in the population. About 20% of all TE copies show transcription in the genome suggesting that genomic defenses such as repressive epigenetic marks and repeat-induced polymorphisms are at least partially ineffective at preventing the proliferation of TEs in the genome. A quarter of recent TE insertions are associated with expression variation of neighboring genes providing broad potential to influence trait expression. We indeed found that TE insertions are likely responsible for variation in virulence on the host and potentially diverse components of secondary metabolite production. Our large-scale transcriptomics study emphasizes how TE-derived polymorphisms segregate even in individual microbial populations and can broadly underpin trait variation in pathogens.

**IMPORTANCE:**

Pathogens can rapidly adapt to new hosts, antimicrobials, or changes in the environment. Adaptation arises often from mutations in the genome; however, how such variation is generated remains poorly understood. We investigated the most dynamic regions of the genome of *Zymoseptoria tritici,* a major fungal pathogen of wheat. We focused on the transcription of transposable elements. A large proportion of the transposable elements not only show signatures of potential activity but are also variable within a single population of the pathogen. We find that this variation in activity is likely influencing many important traits of the pathogen. Hence, our work provides insights into how a microbial species can adapt over the shortest time periods based on the activity of transposable elements.

## INTRODUCTION

Rapid adaptive evolution enables microbial species to cope with challenging environmental conditions including climate change. Most evidence for rapid adaptation comes from experimental studies applying artificial selection pressures (1). How adaptive genetic variation is generated or maintained in natural populations remains poorly explored. Transposable elements (TEs) were recently recognized as key drivers of adaptive genetic variation within species and even single populations (2–4). TEs are genomic sequences that can be mobilized, and high activity in some species was linked to the rapid gain of adaptive variation (5–8). Based on the mode of proliferation, TEs are categorized into retrotransposons, which copy *via* an RNA intermediate, and DNA transposons which can excise and integrate into a different locus. TE-encoded transposase proteins mediate the excision and integration of TEs into the genome (9). TEs create genetic variation in populations through their mobilization. New TE copies can affect gene functions, for example, by insertion into *cis*-regulatory elements or serve as alternative promoters that render a gene more responsive (10, 11). Similarly, TE exaptation into coding sequences can produce novel regulatory sequences and lead to intronization or exonization events with beneficial or detrimental effects for the host (12, 13). Also, TEs can be an important source of epigenetic modifications regulating gene expression (14). Thus, TEs actively inserting into new genomic loci can produce a multitude of effects at the level of gene functions and expression of phenotypic traits (15–17).

TE insertion polymorphisms (TIPs) within species are characterized by individual genotypes differing in the presence or absence of a specific TE at a specific locus. Such insertion polymorphism has well-documented effects in plants such as *Arabidopsis thaliana* (18) and *Oryza sativa* (19). However, TIPs are also widespread in fungal genomes of plant pathogens including *Zymoseptoria tritici* and *Parastagonospora nodorum* (3, 20). The effects of TE insertions segregating within species largely depend on the site of insertion. Due to epigenetic silencing, most TE copies in eukaryotic genomes are not transcriptionally active (21). TE-rich genomic regions carry repressive epigenetic marks, such as histone modifications and DNA methylation. These modifications prevent the transcriptional machinery from accessing TE sequences, thereby silencing TE copies. Beyond epigenetic silencing of TE-rich regions, genomes can encode specific machinery to defend against active transposition. This includes RNA interference, which can bind and cleave transcribed TE sequences (22, 23). In some fungal genomes, defenses expand beyond interference and include repeat-induced point mutations (RIP) as a highly targeted mechanism to counteract TE activity. RIP introduces C->T mutations in repetitive DNA during meiosis and is thought to largely prevent the retention of duplicated sequences in the genome (24).

Assessing the landscape of TEs in the genome helps distinguish between silenced and deactivated TEs from copies with the potential to proliferate. Genomes of fungal pathogens including the plant, animal, and the broad host range pathogens carry large TE-rich regions that also encode virulence-related genes (25–28). The TE-rich regions influence genome plasticity and effector expression in the rice blast pathogen *Magnaporthe oryzae* (29–31). Effector genes in the pathogen *Leptosphaeria maculans* on *Brassica* undergo rapid evolutionary and epigenetic changes near TEs (32). The wheat pathogen *Pyrenophora tritici-repentis* shows higher TE content in pathogenic isolates compared to nonpathogenic isolates, with evidence of TE-mediated effector diversification and movement of virulence factors (33–35). The economically important wheat pathogen *Z. tritici* carries one of the most dynamic fungal genomes with a TE content varying from 16% to 24% among isolates (36). Some TEs share a similar epigenetic niche in the genome as effector genes. Through this co-localization, some TEs and effector genes experience concurrent de-repression during plant infection (37). Silenced TEs near an effector are linked to reduced expression and higher damage on a specific wheat cultivar (37–39). TE insertions upstream of the gene encoding the transcription factor Zmr1 regulate melanin production, a pigment required for pathogen survival during stressful conditions (40). The insertion of a retrotransposon into the promoter of a gene encoding a transporter increases fungicide efflux and contributes to multi-drug resistance (41). Similarly, TE insertions are linked to the adaptive deletion of a gene encoding an effector, which is likely recognized by the plant host (42). The activity of TEs is generally high in the pathogen and varies with geography (3, 36, 43, 44). This is partially explained by the recent loss of genomic defenses against TEs with DNA methylation deactivated at the origin of the species and RIP likely losing efficacy during the colonization of new continents (43, 45, 46).

In this study, we interrogated large transcriptomic, genomic, and phenotypic data sets of a *Z. tritici* population to identify associations between transcriptionally active TEs and adaptive trait variation. We further analyzed variation in TE transcription among individuals and quantified the strength of genomic defenses against TE loci and repressive epigenetic marks. Finally, to capture TE-driven phenotypic variation in the pathogen, we associated metabolite production variation and pathogenicity-related traits of the fungal population with TE insertion polymorphisms.

## RESULTS

### A diverse pool of active TEs in the genome of *Z. tritici*

**Fig 1:**
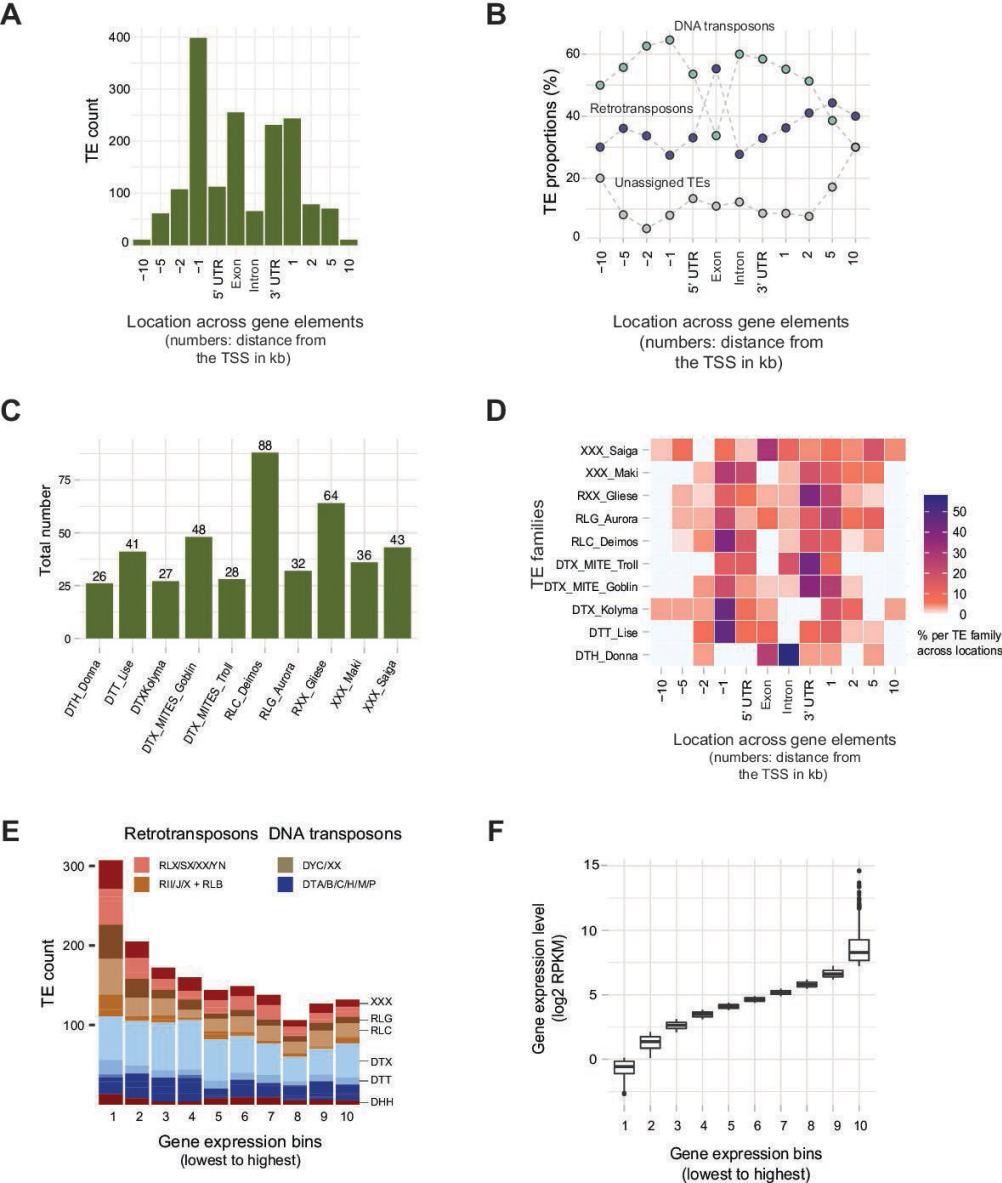
Distribution of transposable elements (TEs) in gene elements in the reference genome of *Zymoseptoria tritici*. (**A**) Genomic localization of TEs (*n* = 1,640) in gene elements and 10 kb windows upstream and downstream of the transcription start site (TSS). (**B**) Distribution of DNA and retrotransposons across gene elements and 10 kb windows upstream and downstream of the gene (887 DNA transposons, 597 retrotransposons, 156 unclassified elements). The dotted lines represent the distribution of TE classes. Exons and introns are grouped into a single category per gene. (**C**) A total number of major TE families (with ≥25 copies in the genome) inserted into gene elements. (**D**) Distribution of the 10 most abundant TE families in gene elements and 10 kb windows upstream and downstream of TSS. Percentages refer to the portion of element copies located in particular gene elements. (**E**) Count of TEs localized close to genes categorized according to 10 bins of gene expression. The weakest expressed genes co-localized with the highest number of TEs. (**F**) Variation in gene expression within each of the 10 gene expression bins.

The TEs in the *Z. tritici* genome were previously identified by screening copies of consensus sequences in a panel of 19 reference-quality genomes to maximize the discovery of low-copy TEs (36). Consensus sequences were defined for TE copies with an identity >80% and >80% length covered. TEs were classified based on protein functions encoded by open reading frames. Short non-autonomous TEs were classified separately (36). We assessed the strength of selective constraints against TEs by analyzing the distribution of TE families relative to gene elements (Table S1 at https://doi.org/10.5281/zenodo.10020096). Based on the telomere-to-telomere assembled reference genome IPO323, we found that ~10% (*n* = 398) of TEs in the genome were located within 1 kb upstream of coding regions likely overlapping with regulatory elements (Fig. 1A). Similarly, exons (6%), 3′ untranslated regions (UTRs, 6%), and downstream regions (<1 kb, 10%) also carried high proportions of TEs near genes. Introns and 5′ UTRs showed low TE counts (1% and 2%, respectively; Fig. 1A). Across gene elements, only few DNA transposons inserted into exons, while retrotransposons made up the highest proportion (Fig. 1B). To investigate the distribution of major TE families across gene elements, we focused on the most abundant TE families (≥25 copies in the genome) including five DNA transposons, three retrotransposons, and two unassigned TE families (Fig. 1C and D). We found that 56% of the copies of the DNA transposon *Harbinger* (DTH) *Donna* were integrated into introns. The tight association of *Donna* with some intron sequences explains previous findings of identical intron sequences in unrelated genes (47). Similarly, more than half of the copies of the DTT_Lise (Tc1-*Mariner* superfamily) and a *Ty1/Copia* element (RLC *Deimos*) were integrated into upstream regions of genes (Fig. 1C). TE families *Gliese*, the MITEs *Troll,* and *Goblin* showed the highest insertion proportions in 3′ UTRs. We next analyzed associations of TE insertions and gene expression levels under nutrient-limited conditions. TEs inserted most frequently close to the 10% lowest expressed genes and the insertion events were dominated by retrotransposons (Fig. 1E). *Ty3/mdg-4* retrotransposons and unassigned TEs show the strongest skew toward integration close to lowest expressed genes (Fig. 1F; Fig. S1). On the contrary, DNA transposons are less depleted near strongly expressed genes. Overall, the TE distribution in the genome is strongly correlated with the transcriptional landscape of genes.

### TE insertion dynamics in a field population

**Fig 2:**
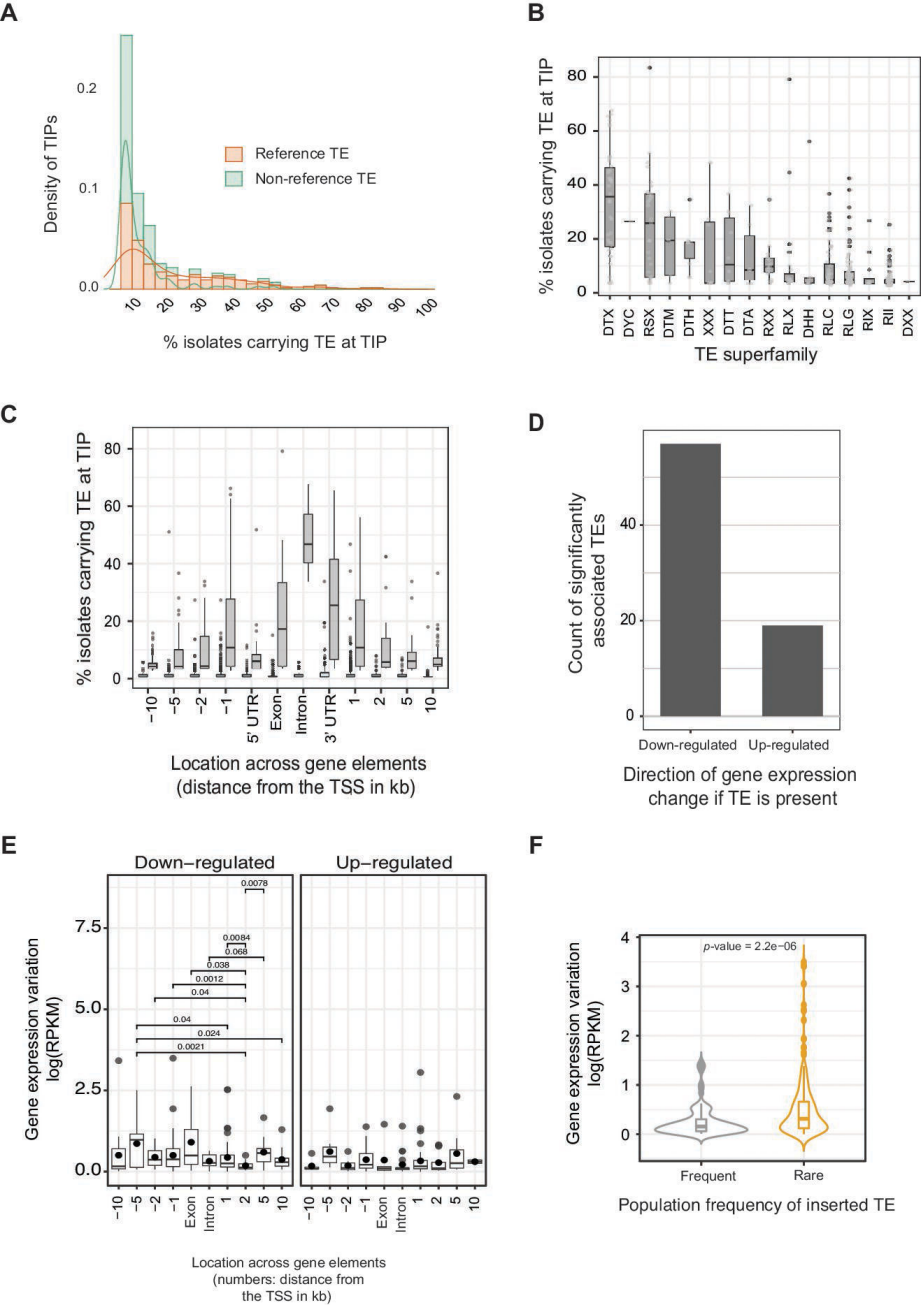
TE insertion polymorphisms (TIPs) in a single field population and association with gene expression variation. (**A**) Frequency of TEs present in the reference genome (“reference TEs”) or only detected in other isolates (“non-reference TEs”). A total of 3,662 non-reference TEs and 446 reference TEs were analyzed. (**B**) The frequency of the inserted TE at TIP loci is expressed as the percentage of the isolates carrying the TE. TIPs were binned by TE superfamilies for visualization. (**C**) The frequency of the inserted TE at TIP loci is expressed as the percentage of the isolates carrying the TE binned by location across gene elements (±10 kb window). (**D**) Count of significantly associated TIPs with neighboring gene expression variation. The classification is based on the up- or downregulation of the neighboring gene if the TE is present. (**E**) Association of inserted TEs at TIPs with the down- or upregulation of the neighboring gene shown for TIPs located in different gene elements. Significant associations are shown with the respective *P*-value. (**F**) Gene expression variation of genes with a neighboring TIP according to the frequency of the inserted TE. TIPs with rare TE presence (<20 isolates; <15%) are significantly associated with genes showing higher gene expression variation. The statistical test is robust to the exclusion of outliers (values > 1.5).

TEs in the species show recent activity and contribute to genome-wide polymorphism (44). We examined TE-generated polymorphism based on TIPs for a deeply sampled single field population of genetically diverse isolates (Table S2 at https://doi.org/10.5281/zenodo.10020096). We used *ngs_te_mapper* to scan 139 whole-genome sequencing data sets individually aligned to the reference genome. TEs absent in the reference genome were rare in the population (“non-reference TEs”; on average carried by 5% of all isolates) in contrast to TEs with a copy present in the reference genome (“reference TEs”; Fig. 2A). This is consistent with the expectation that TEs present in the reference genome are more likely to be at a higher frequency among other isolates as well. DTX is the most frequent TE superfamily showing TIPs in the population followed by SINE (RSX) and mutator elements (DTM; Fig. 2B). TIPs inserted into introns had the highest population frequency compared to TEs inserted in other gene elements (Fig. 2C). For example, 46% of isolates contained intron insertions of the TIR DTX elements. Furthermore, 79% of isolates from the population carried LTR elements (RLX) in exons (Fig. S2). Given the high numbers of TIPs near coding sequences, we analyzed associations of TIPs with gene expression (Table S3 at https://doi.org/10.5281/zenodo.10020096). We analyzed genes with TIPs showing a minor allele frequency >5% and being located within 10 kb of the gene. We compared transcript levels between isolates with and without TE insertions for a total of 354 TIPs. Approximately 21.4% (*n* = 76) of the TIPs in the population showed a significant association with the expression of a neighboring gene (*P*-value < 0.05). Overall, the majority of TE insertions near a gene tend to reduce the gene transcription levels (Fig. 2D). Interestingly, the distance at which the TIP is located compared to the gene with associated gene expression variation seems not to be an important factor determining the extent of gene expression variation (Fig. 2E). Next, we analyzed the population frequency of the inserted TEs and gene expression variation among isolates. We found that rare TE insertions at the population level were significantly associated with higher expression variation of neighboring genes (Fig. 2F) and the association is robust to the removal of outliers with log (RPKM) >1.5. In summary, the genomic landscape of polymorphic TE insertions reflects gene expression variation within the pathogen population.

### Locus-specific transcription of TEs

**Fig 3:**
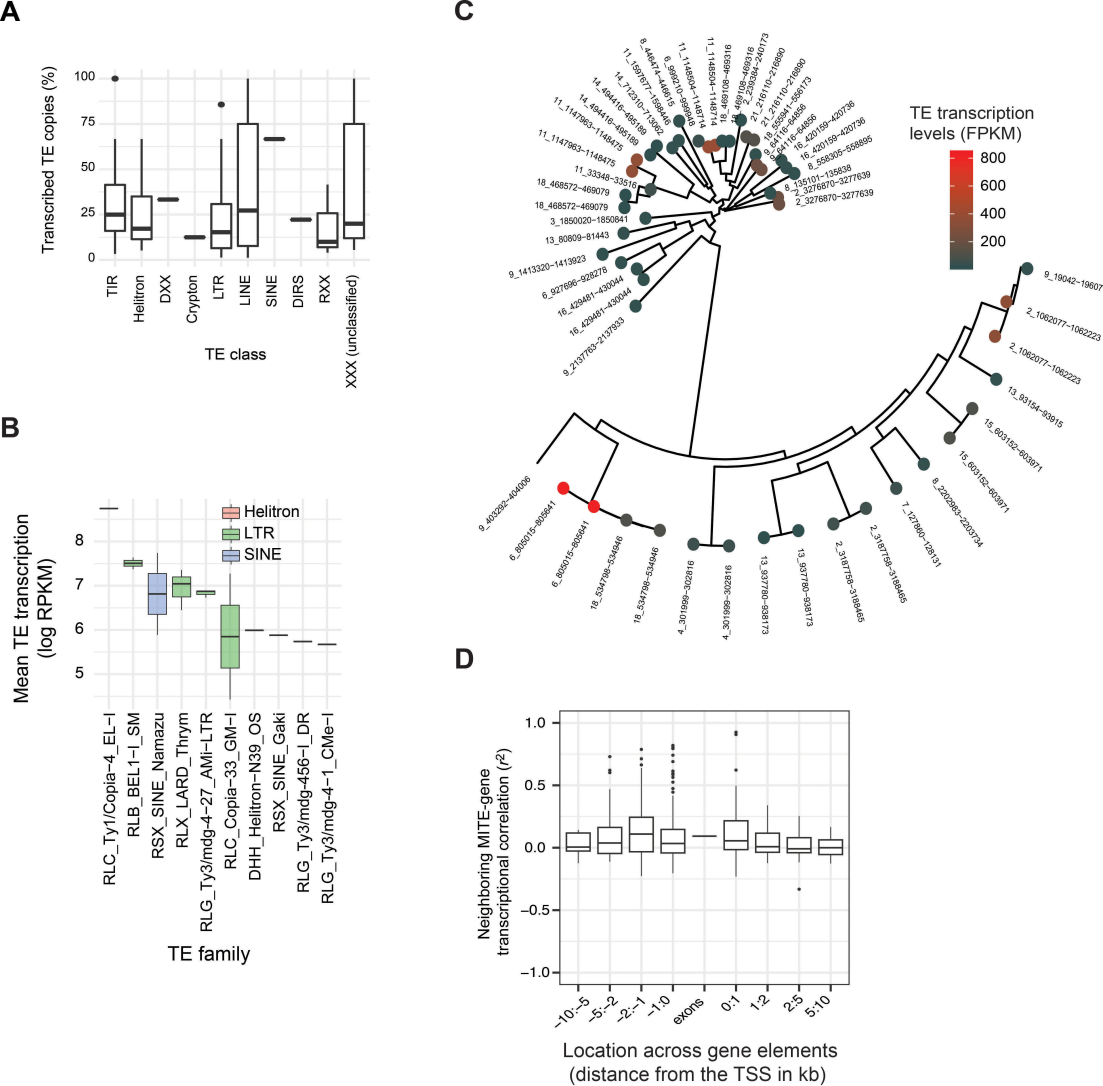
Population-level variation of locus-specific transcription of TEs. (**A**) Percentage of transcribed TE copies per TE family grouped by TE superfamily. (**B**) Transcription levels of individual copies of the ten most transcribed TE families in the genome. (**C**) Neighbor-joining tree of all copies of the MITE *Undine* family discovered in the IPO323 reference genome. Colors indicate transcription levels for each copy. (**D**) Correlation of transcription between MITE copies and neighboring genes. The correlation values are summarized based on the distance of the MITE from the TSS.

Transcription of TEs can lead to transposition and copy number increases depending on the type of TE. To identify the pool of transcribed TEs from each TE family, we analyzed poly-A tail enriched RNA-seq data. We generated locus-specific expression estimates based on uniquely mapping RNA-sequencing reads to identify variation in transcriptionally active copies of the same TE family (Table S4 at https://doi.org/10.5281/zenodo.10020096). Reads mapping to multiple loci of the same TE family were distributed proportionally across loci according to the algorithm to account for relative differences in transcriptional activity among TE families. We found that 43% of all TE copies per genome show evidence of transcription and substantial variation among individual TE families and superfamilies (Fig. 3A; Fig. S3). We observed the highest transcription levels for LTRs including RLB_BEL1, RLX_LARD, and RLG *Ty3/mdg-4* (Fig. 3B). Although the number of TE copies significantly differs among chromosomes, we found no significant variation in transcription (Fig. S4). Sequence similarity of recently transposed TE copies can be a challenge for the unique mapping of sequencing reads to individual loci. Hence, locus-specific analyses are likely biased against the youngest TEs (identified by the presence of near-identical copies) in the genome. To assess this risk, we reconstructed the phylogenetic tree of an MITE family using sequences of individual TE copies in the genome. We assessed transcript abundance for each locus in association with the terminal branch length of the individual copy. In general, short branch lengths indicate rapid expansion of copies. We found no meaningful association between the sequence similarity of copies of a TE family and transcript abundance suggesting that expression quantification introduces no major bias against young TEs as illustrated for a MITE family (Fig. 3C) and an RLX_Lard_Gridr (Fig. S5). Overall, transcription of MITE copies near genes tends to be positively correlated with the expression of genes, in particular at distances of 0–2 kb from the TSS (Fig. 3D) suggesting shared epigenetic effects.

**Fig 4:**
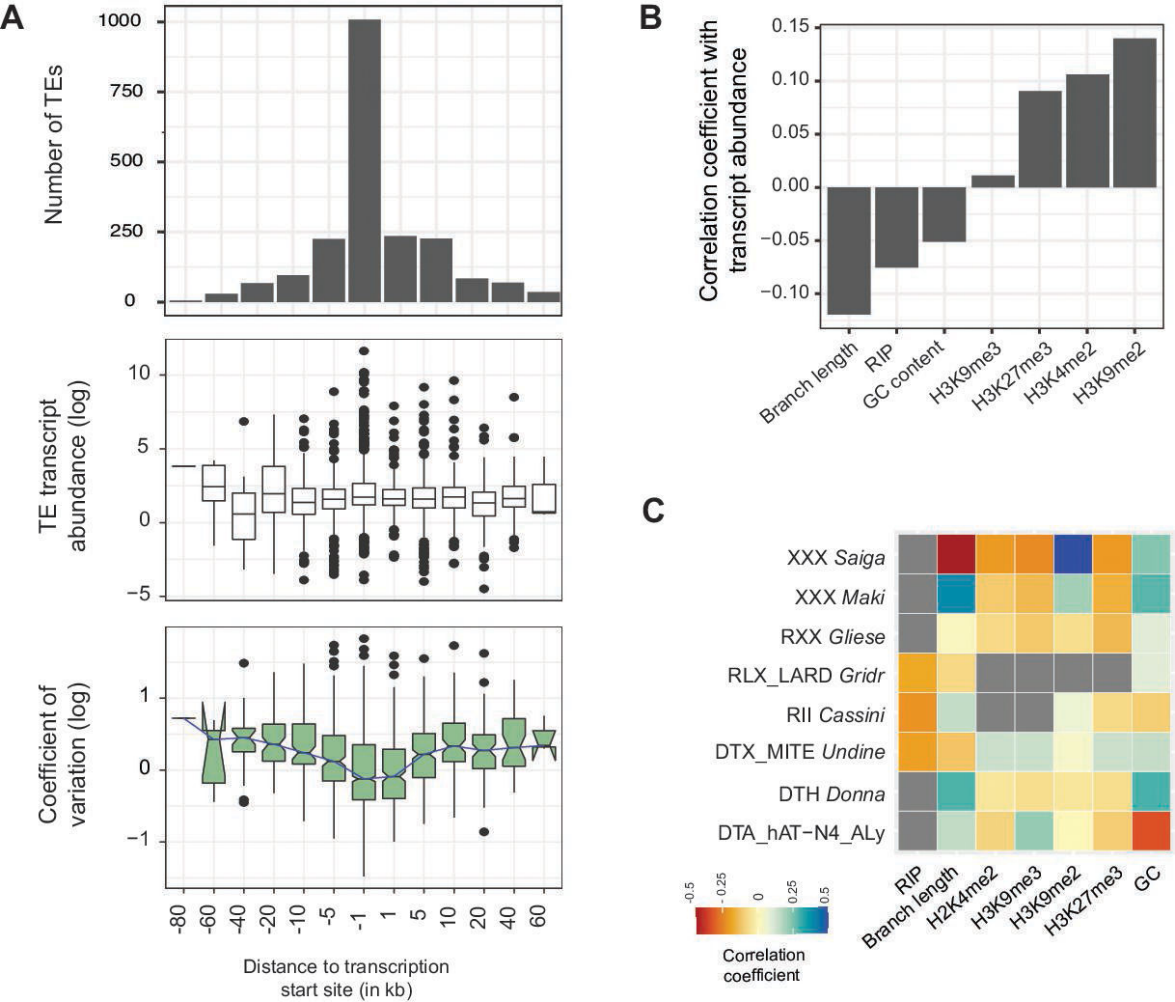
Transcriptional landscape of TEs across the genome. (**A**) Number of TEs, transcript abundance, and coefficient of variation of TEs localized upstream and downstream of the TSS of a gene (number of TEs = 2083). (**B**) Correlation of transcript abundance detected at individual TE loci and branch length of the TE copy compared to other TE copies of the same family, the number of RIP mutations, GC content, and different histone methylation marks (assessed for *n* = 455 TE loci). (**C**) Analyses of the eight most transcribed TEs (>25 transcribed copies each). Correlation matrix of transcription of individual TE loci and genomic features as shown in (B).

The genomic context and the identity of individual TEs are potentially significant factors explaining genome-wide TE transcription and insertion activity. First, we found that the highest number of TEs were located within 1 kb upstream of the gene transcription start sites (Fig. 4A). We also found that TE copies further away from the TSS tend to show higher transcriptional variation among isolates compared to TE copies < 1 kb from genes but at comparably transcription levels (Fig. 4A). Epigenetic factors such as histone methylation marks including H3K4me2, H3K9me2, H3K9me3, and H3K27me3 can have important effects on gene and TE transcription (48, 49). In addition, repeat-induced point (RIP) mutations, and GC content of TE loci and their association with transcription can be important indicators of the activity of TEs (24, 50). We found a negative correlation between TE transcription levels and the phylogenetic distance among TE copies of the same family (i.e., branch length) as well as counts of RIP mutations. This is consistent with the action of RIP as a genomic defense against TEs. Consistent with expectations, euchromatic histone methylation marks such as H3K4m3 were positively correlated with the transcription of TEs. Surprisingly, also repressive histone methylation marks (H3K27m3 and H3K9m2) were positively correlated with TE transcription (Fig. 4B).

Analysis of correlates among genomic features and TE transcription at the level of individual TE families revealed a more complex landscape of associations compared to the global level of all TEs combined (Fig. 4C). The non-autonomous TE family DTX MITE *Undine* showed a negative correlation between transcription and the phylogenetic distance of the TE copy (using terminal branch lengths) and a slightly positive correlation with both repressive and euchromatic histone methylation mark (Fig. 4C). The uncharacterized TE *Saiga* showed both a strong negative correlation with branch length of the copy and a strong positive with GC content and repressive histone methylation marks. We further investigated interactions of the genomic environment with TE transcriptional activity. We found a positive relationship between the proportion of transcribed copies of a TE in a genome with the percentage of isolates carrying expressed TE copies. For instance, among DNA transposons, the MITE *Undine* had more than 50% of the copies expressed in the reference genome and more than 75% of the Undine copies were transcribed among isolates. For the DTA *hAT* element, less than 30% of the copies were expressed in the genome as well as among isolates (Fig. S6; Table S5 at https://doi.org/10.5281/zenodo.10020096).

### Enrichment of repeat-induced polymorphisms in regulatory regions of TEs

**Fig 5:**
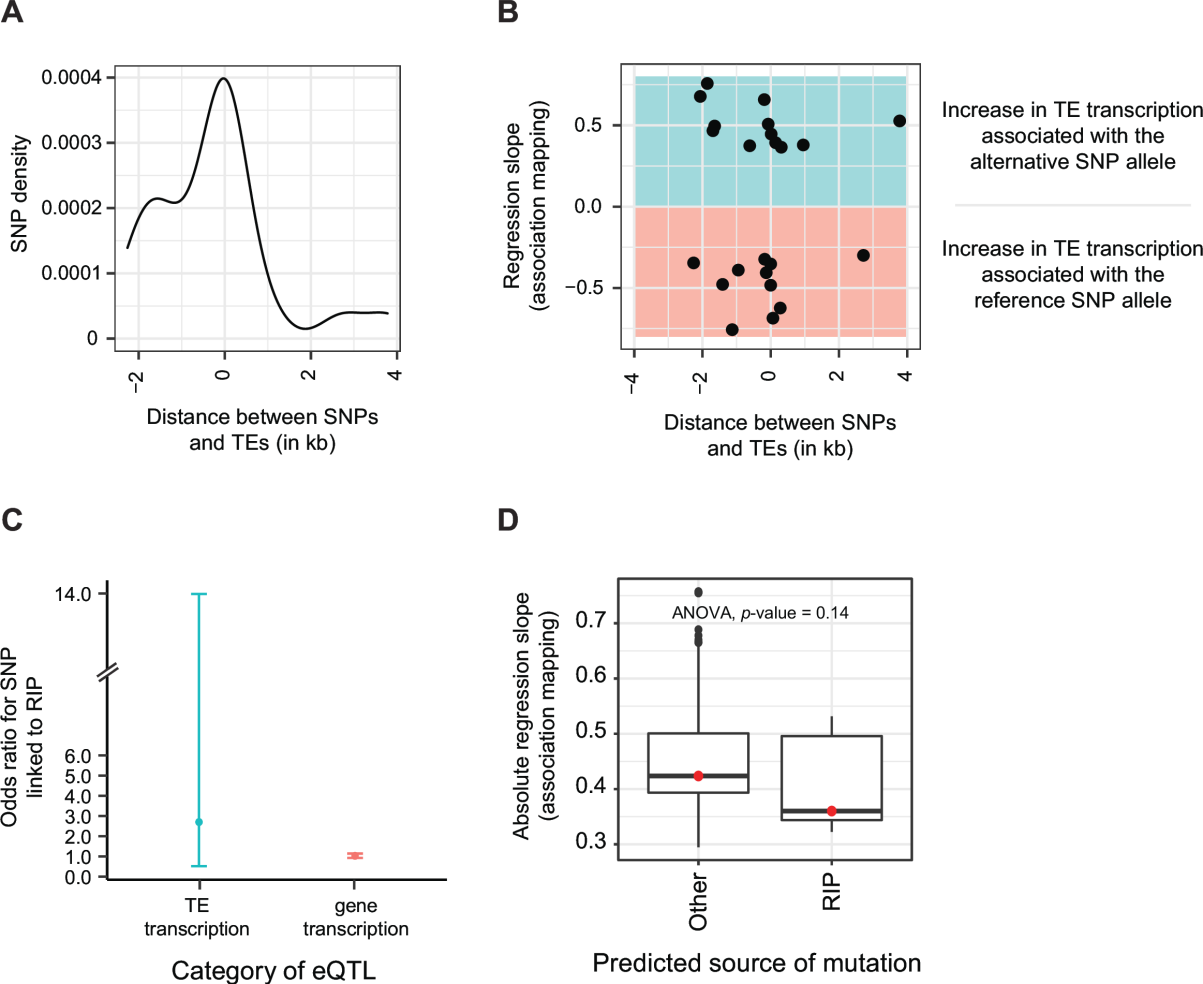
Regulatory variants associated with TE transcription. (A) Distribution of SNPs associated with TE transcription variation among isolates. The majority of the significant SNPs are located within a 2 kb distance upstream and downstream of TE loci (*n* = 22). (B) Regression slope (i.e., effect size) of significantly associated SNPs with TE transcription variation (for *n* = 22 associated SNPs). The colors represent negative and positive regression slopes highlighting either a positive expression association with the reference or an alternative allele at the SNP. (C) Enrichment of RIP-like mutations in SNPs associated with TE transcription variation compared to SNPs associated with gene expression variation. An odds ratio >1 indicates an enrichment of RIP-like mutations and 95% confidence intervals are shown. A total of 197 SNPs were analyzed for an association with TE transcription variation and 283,867 SNPs were analyzed for an association with gene expression variation. (D) Absolute regression slope (i.e., effect size) of RIP-like vs other SNPs associated with TE transcription variation (for *n* = 22 associated SNPs).

We used genome-wide association mapping to systematically identify single nucleotide polymorphisms (SNPs) associated with variation in TE transcription among isolates. We associated SNPs within a 5 kb distance both upstream and downstream of the TE with the transcriptional variation of the TE in the population. We found at least one significantly associated SNP in proximity to 22 TE loci with nearly all significant SNPs located within 2 kb of the TE (Fig. 5A). Regression slopes for transcriptional variation association mapping are positive if the alternative allele is associated with higher transcription. A negative slope stems from higher transcription associated with the reference genome allele. We found no meaningful effect of reference vs alternative SNP allele or distance of the SNP to the TE (Fig. 5B). Next, we assessed whether there was an enrichment for RIP-like mutations among TE transcription-associated polymorphisms. In *Z. tritici*, RIP induces C→T transitions at CpA sites (45). We analyzed all SNPs in the genome associated either with TE transcription or gene transcription (51). Then, we assessed whether the polymorphism is likely RIP associated. We found that for TE transcription-associated SNPs, the odds ratio of the SNP being caused by RIP was 2.70 (CI 0.52–13.98; Fig. 5C). In contrast for gene transcription-associated SNPs, the odds ratio of the SNP being caused by RIP was only 1.03 (CI 0.93–1.14). Given the overlapping confidence intervals, it remains unclear whether RIP plays a more pronounced role in generating SNPs governing TE transcription variation rather than gene transcription. Finally, we analyzed whether the strength of the TE transcriptional association expressed by the regression slope differs depending on whether the SNP was likely caused by RIP or not. We found no significant differences in regression slopes depending on the source of the mutation (Fig. 5D).

### Phenotypic variation associated with recent TE insertions in a population

**Fig 6:**
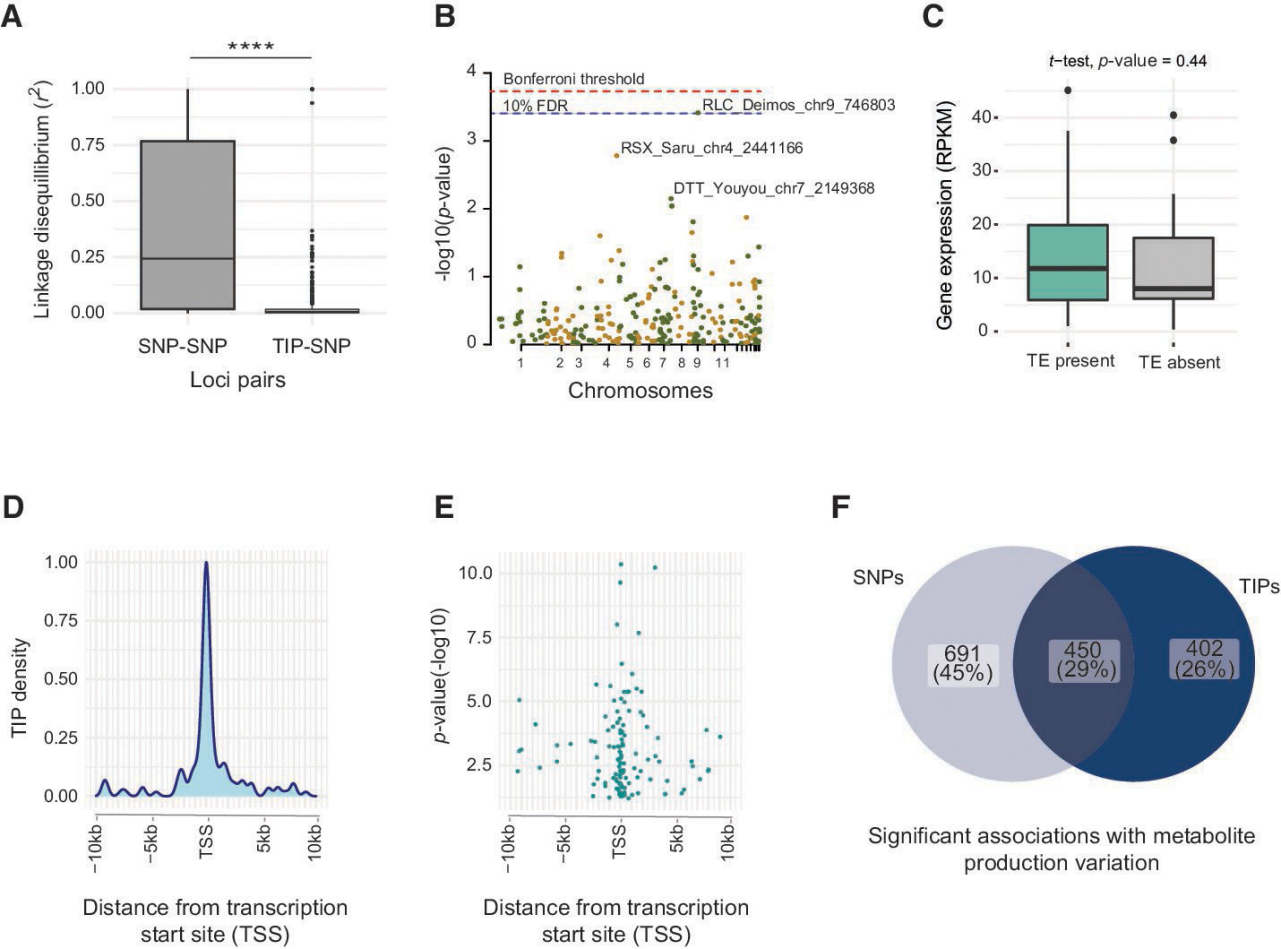
Phenotypic-genotype associations at TIP loci. (**A**) Linkage disequilibrium (LD) between TIPs and SNPs in 5 kb windows as well LD of SNP-SNP pairs in the same windows (number of SNPs in analyzed TIP windows = 12,832). (**B**) Manhattan plot for TIP association mapping with virulence (measured by the percent leaf area covered by lesion). Colors refer to TIPs located on distinct chromosomes. (**C**) Gene expression variation of a gene encoding a cell wall-degrading enzyme according to the presence or not of a TE at the TIP (*t*-test, *P*-value = 0.44). (**D**) Distribution of TIPs (*n* = 1213) is significantly associated with variation in metabolome peak intensities near transcription start sites (TSS) of genes. (**E**) *P*-value distribution of TIPs associated with metabolome peak intensities near the TSS of genes. The most significantly associated TIPs are located closest to the TSS. (**F**) Venn diagram representing the overlap of significant association from TIP metabolome GWAS and SNP-based metabolome GWAS.

Insertions caused by TEs can have consequences for the expression of phenotypic traits. However, a range of additional genetic variants are likely to impact trait variation as well. To assess the likelihood of TE loci influencing trait expression independently of other polymorphisms, we analyzed patterns of linkage disequilibrium (LD) between TIPs and nearby SNPs. We found that most TIPs were on average in low LD with SNPs within a 600 bp window compared to LD between pairs of SNPs in the same window (Fig. 6A; Table S6 at https://doi.org/10.5281/zenodo.10020096). However, SNPs and TIPs tended to be at a longer distance within the window compared to SNP-SNP distances. This phenomenon is likely to explain the low LD observed between SNPs and TIPs (Fig. S7). To identify associations of TIPs with phenotypic trait variation, we analyzed a series of trait data sets generated for the same population including pathogenicity trait measurements and variation in metabolite production (Table S7 at https://doi.org/10.5281/zenodo.10020096). Wheat leaf lesion area produced by individual isolates showed an association significant at the 10% false discovery rate (FDR) threshold with a TIP caused by a 6 kb *Ty1/Copia* element (RLC *Deimos*) located on chromosome 9 (Fig. 6B). The associated TIP is within ~5 kb of a gene encoding a cell wall-degrading enzyme (i.e*.,* glycosyl-hydrolase family 47) secreted during host infection. We found no significant difference in the expression of the gene encoding the cell wall-degrading enzyme between isolates carrying the *Ty1/Copia* element or not (*t*-test *P*-value = 0.44; Fig. 6C).

Given the large scope for TE insertions associated with gene transcription variation, we screened the population for additional phenotypic readouts. We analyzed a data set of metabolite production profiles of each isolate assessed under culture conditions (Table S8 at https://doi.org/10.5281/zenodo.10020096) (52). We found that 65% of all TIPs were significantly associated with variation in intensity in at least one metabolite (Table S9 at https://doi.org/10.5281/zenodo.10020096). Out of the significantly associated TIPs, 13 were significantly associated with at least 20 different metabolites (Fig. S8). We found that TIPs located within ~1 kb upstream of the TSS of genes have a stronger association with variation in metabolite production than TIPs further away from the TSS (Fig. 6D and E). The enrichment of significant TIPs within coding regions and near TSS is consistent with the observations from a metabolome GWAS (52) performed on the same study population using genome-wide SNPs. Overall, 26% of all significant associations with individual metabolite production variation using TIP-GWAS were not previously identified using SNPs only (Fig. 6F).

**Fig 7:**
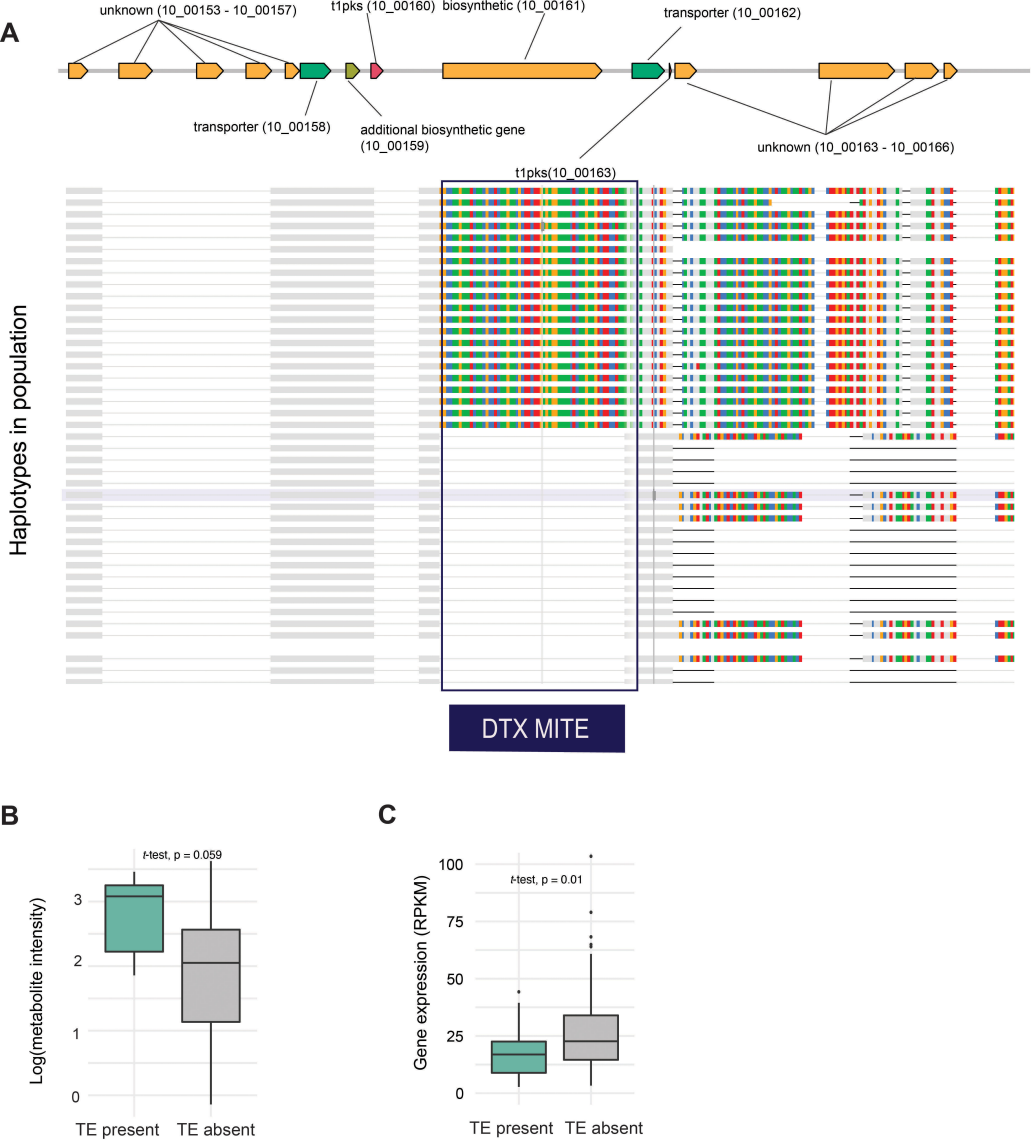
Genomic context of the MITE insertion in a secondary metabolite gene cluster. (**A**) The top drawing shows the genomic organization of the PKS1 secondary metabolite gene cluster. Genes are labeled with functional annotation and genomic location. The multiple sequence alignment highlights the conserved MITE insertion shared among isolates (i.e., haplotypes). (**B**) Association of metabolite production intensity with the MITE presence/absence variation. (**C**) Association of the *NmrA*-like gene expression variation associated with the MITE presence/absence variation. Number of isolates with TE present = 17 and isolates with TE absent = 129.

Based on the metabolite-TIP GWAS, we identified a significant association for a DTX MITE insertion with the metabolite profile *m/z* intensity 245.1866. The associated MITE insertion is in the intergenic region of a polyketide synthase (PKS) secondary metabolite gene cluster located on chromosome 10 (position 427,490–477,261 bp) associated with higher metabolite production (Fig. 7A). The insertion occurred upstream of the gene encoding a NmrA-like family protein (Zt09_10_00163). NmrA is a negative transcriptional regulator involved in nitrogen metabolite repression in pathogenic fungi (53). We found that the MITE insertion was significantly associated with lower expression of the NmrA-like family protein (Fig. 7B and C).

## DISCUSSION

We performed a comprehensive analysis of transcriptional variation generated by individual TEs in a pathogen population and consequences for phenotypic trait evolution. Recent TE insertions generated presence-absence variation among isolates (i.e*.,* TIPs) with selection likely playing an important role in filtering new insertions. Beyond the single field population, the species contains multiple, highly active TEs with diverse effects on trait expression, epigenetic variation, and even genome size (37, 44, 54). We identified considerable intraspecific variation in the transcription of individual TE copies emphasizing the importance of the genomic background for controlling TE transcription. Finally, TE insertions likely influence the gene expression of neighboring genes and the expression of traits including metabolites, which can mediate organismal interactions.

We found approximately a fifth of all TE copies in the genome with transcriptional activity. This indicates significant potential for TE mobility depending on the mode of proliferation. Assessing locus-specific transcription is challenging though given the repetitive nature of TEs. Our approach was based on short RNA-seq reads uniquely mapped to specific TE loci in the genome (55). However, not all TE-derived RNA-seq reads can be assigned using this restrictive approach. All non-uniquely mapped TE-derived reads are distributed proportionally among TE loci of the same family. This latter step enables accurate quantification of TE transcription among TE families at the detriment of accurate TE quantification among TE loci of the same TE family. Long-read transcriptome sequence (e.g., Iso-Seq) largely solves the challenge of unique read mapping; however, such approaches come at the expense of coverage and, hence, quantification accuracy. The compact genome of *Z. tritici* (~40 Mb) and comparatively low TE content (16%–24%) facilitates accurate read mapping compared to large animal genomes for which the approach was initially developed (55).

A high number of TE insertions are not fixed within the population suggesting ongoing activity creating new copies. However, some segregating TE insertions may be old, and estimating the age of an insertion is difficult without access to allelic variants of an inserted TE and surrounding sequences. If purifying selection is strong though, TE insertions should remain at low frequency and tend to be young as shown by a global TE analysis of *Z. tritici* populations (3). Consistent with strong selection, TE insertions were more likely to occur in non-coding regions than exon or intron sequences carrying a high likelihood of deleterious effects. We also found that the identity of TEs is an important factor explaining their distribution across gene elements with, for example, some TEs nearly exclusively associated with intronic insertions. Some introns in *Z. tritici* were identified as non-autonomous TEs because unrelated genes across the genome harbor nearly identical intron sequences (47, 56). Overall, the species harbors dozens of polymorphisms for the presence-absence of entire intron sequences consistent with the recent mobility of intronic sequences (47). Retrotransposons were overrepresented in exonic sequences compared to DNA transposons. This may be explained at least partially by gene models including open reading frames of TEs or chimeric genes. TE frequencies at TIPs tend to be low except the intronic TEs, which are most likely domesticated with no or only minor deleterious effects. Overall, the TE landscape of a single population likely largely reflects insertion constraints and counter-selection against TE insertions.

Active transcription of a TE can generate new TE insertions in the genome depending on the mechanism of propagation, as well as impact the structure and function of the genome (15, 57). Selection acting against deleterious TE insertions is complemented by specific genomic defense mechanisms that evolved to counteract TE activity in fungal genomes. These mechanisms include RIP mutations acting against duplicated sequences and epigenetic silencing reducing the transcriptional activity of TEs and accessibility of DNA (14, 21, 24). We found mostly low to no transcription of TE copies affected by RIP suggesting that the defense mechanism effectively silences TEs similar to *N. crassa* (50). The most abundant TE family (i.e., the MITE *Undine*) showed the highest transcriptional variation for individual loci in the population. The same TE is also highly upregulated during plant infection and has undergone a massive recent expansion (37, 44). Hence, variation in MITE transcription near coding sequences likely reflects epigenetic variation. As MITEs are non-autonomous elements relying on transposition functions provided by full-length TEs elsewhere in the genome, transcription of MITEs is most likely uninformative about their potential to spread in the genome. Recapitulating MITE mobilization would require analyses at the population level to match MITE sequence characteristics (i.e., terminal sequences or target site duplications) to full-length TEs potentially serving as vehicles for MITE transposition. The weak effect of genomic defenses against MITEs is also likely related to the short length (~53 bp), which renders RIP-based defenses ineffective (20, 37). The heterogeneous effects of RIP against different classes of TEs are compounded by the relaxation of RIP genomic defenses within the species following the expansion from the center of origin in the Middle East (43, 45, 58).

Transcription and distribution in the genome were highly uneven among TE families consistent with differences in recent activity and insertion preferences matching evidence from other kingdoms (59). The *Mutator* elements of maize typically integrate into gene-rich regions (60). In *S. cerevisiae*, *Ty1/Copia* integrates preferentially upstream of genes transcribed by the RNA polymerase III (Pol III) (61, 62). *Ty1* is also enriched in heterochromatic regions triggered by the recognition of heterochromatin during integration and then perpetuates the heterochromatic mark by triggering epigenetic modifications at new insertion loci (63). The *P* elements in *Drosophila* preferentially integrate at replication origins in the genome to support transposition (64). We also find that TE insertions are underrepresented near highly expressed genes similar to the observation in *D. nasuta* (65). Those observations may however be the result of a TE survivor bias given the typically slow evolutionary rates of highly expressed genes given the strong purifying selection (65, 66). Under some conditions, the spread of heterochromatin from TE insertion sites can induce epigenetic silencing of neighboring genes (14). Consistent with this possibility, we found that TE insertions are significantly associated with expression variation of neighboring genes. TE insertion tends to be associated with lower gene transcription levels consistent with previous analyses of gene expression analyses over the course of plant infections (37). We found surprising positive associations between repressive heterochromatin and increases in TE transcription. Without access to histone methylation data at the population level, interpreting potential mechanistic links between the two factors is challenging. It is important to note also that histone methylation data were collected in less stressful environmental conditions than the TE expression quantification. Based on environmental cues and stress induction, TEs show distinct de-repression patterns throughout an infection with correlated responses of genes in proximity (67). Our findings expand our understanding of the coordinated expression of TEs and adjacent genes by showing that these patterns can be driven by polymorphic TE insertions.

Crucial for the adaptive evolution of microbial species, TE insertion activity in genomes can drive phenotypic trait evolution through their impact on gene structure and regulation. We showed that variation in pathogenicity-related traits and metabolite production is most likely underpinned by recent TE insertion activity in functionally important regions of the genome. Our TIP-GWAS showed that two-thirds of all TIPs showed a significant association with at least one metabolite intensity profile. The specific biological roles of the different metabolites are poorly understood with few exceptions (52, 68). However, individual metabolites can play major roles in species interactions with competitors or hosts (40, 69). Similarly, TE insertions in crop plants are associated with a wide range of agronomic traits and secondary metabolites such as in tomatoes (4, 70). Metabolite production-associated TIPs in *Z. tritici* showed enrichment in coding regions and transcription start sites consistent with these regions having a higher potential for functional consequences. Given the large number of TE insertions associated across many genes for metabolite production provides a vast potential for rapid evolution of the species from standing variation in single field populations. TE-driven adaptation within species and populations is likely to proceed at a more rapid pace given the potential for stronger phenotypic variation among genotypes at TIPs. Consistent with individual findings across plants, animals, and fungal species, active TEs in the genome can underpin the most recent adaptive evolution of large effect size. The power of TEs to drive evolutionary change stems from their potential to affect the expression of multiple nearby genes, modulate their response to external stress but also to rearrange large sequence segments including the deletion and duplication of gene sets. Recent discoveries of large TEs with the ability to conjugate dozens of genes and hundreds of kilobases of sequences (71) exemplify the driving force transposable elements play underpinning the rapid evolution of microbial species.

## MATERIALS AND METHODS

### Fungal isolates and sequencing

We analyzed *Z. tritici* isolates collected from a wheat field in Eschikon, Switzerland. Wheat cultivars were planted in a randomized block design and infections occurred naturally from local inoculum or adjacent fields (72). To isolate strains, individual cirri from pycnidia were identified on infected leaves and plated on a yeast sucrose broth (YSB) solid media plate with kanamycin 50  µg mL^−1^ and incubated at 18 °C. After a week, a single colony from each plate was inoculated in a yeast-sucrose broth (YSB) and incubated on a shaking incubator at 18°C for 8  days at 140–180  rpm (72). Total genomic DNA was extracted from 139 *Z. tritici* isolates YSB cultures using the QIAGEN DNAeasy Plant Mini Kit. Illumina libraries were prepared using the TruSeq Nano DNA Library Prep kit. Sequencing was performed in 100 bp paired-end mode on a HiSeq 4000 at the iGE3 sequencing platform (Geneva, Switzerland). Raw reads are available on the NCBI Short Read Archive under the BioProject PRJNA596434. For RNA sequencing, the same isolates (146 isolates were cultured in a Vogel’s Medium N (Minimal) modified as ammonium nitrate replaced with potassium nitrate and ammonium phosphate (73) without sucrose and agarose to induce hyphal growth. Total RNA was isolated from the filtered mycelium after 10–15 days using the NucleoSpin RNA Plant and Fungi kit. The RNA concentrations and integrity were checked using a Qubit 2.0 Fluorometer and an Agilent 4200 TapeStation system, respectively. Only high-quality RNA (RIN >8) was used to prepare TruSeq-stranded mRNA libraries with a 150 bp insert size and including a poly-A enrichment step. Sequencing was performed on an Illumina HiSeq 4000 in 100 bp single-end mode. Raw reads for RNAseq are available on the NCBI Short Read Archive under the BioProject PRJNA650267 (52; Table S10 at https://doi.org/10.5281/zenodo.10020096).

### Identification of TE insertion polymorphism in *Z. tritici*

Whole-genome Illumina sequencing reads were quality checked using FastQC [version 0.11.5 (74)] and trimmed with Trimmomatic version 0.36 (75) to remove adapter sequences and low-quality reads with parameters ILLUMINACLIP: TruSeq3-PE.fa:2:30:10 LEADING:3 TRAILING:3 SLIDING WINDOW:4:15 MINLEN:36. To detect TE insertions, we used the R-based tool *ngs_te_mapper* version 79ef861f1d52cdd08eb2d51f145223fad0b2363c (76) integrated into the McClintock pipeline version 20cb912497394fabddcdaa175402adacf5130bd1 (77). Filtering and validation of the identified insertion polymorphisms followed previously established protocols for the same species (3). To confirm the presence of predicted non-reference TEs, we extracted the reads mapped near the predicted insertion site and assessed whether the target site duplication represented a break in the alignment as expected. Using spliced junction reads, we analyzed whether a gap was suggested in the region of the position indicating the absence of a reference genome TE copy in a particular isolate.

### Locus-specific TE and gene expression analyses

Locus-specific expression profiles for TEs were generated using mapped RNA-seq data with the tool SQuIRE (v0.9.9.9a-beta) (55). TE annotation of the reference genome IPO323 was retrieved from (36). The SQuIRE “Map” mode was used to align RNA-seq data and SQuIRE “Count” with the –EM parameter to perform the estimation-maximization algorithm to quantify TE expression considering both uniquely mapped and multi-mapped reads typically found in repetitive sequences such as TEs. TE-derived reads normalized by fragments per kilobase of exon per million (FPKM) were filtered to keep the reads originating from the annotated strand direction in the IPO323 reference genome. Locus-specific expression analyses can be influenced by high sequence similarity among copies in recently expanded TE families. To assess the impacts of high sequence similarity among copies, we have generated a phylogenetic tree of the MITE family *Undine* using individual locus sequences. Branches were annotated with corresponding transcript abundance. We used neighbor-joining tree estimation (njs) in the R (78) package *ape* (version 5.5) (79) and the transcript abundance was plotted using the R package *ggtree* (version 3.0.4) (80). In addition, gene expression profiles were analyzed using QTLtools (version 1.1) (81). RNA-seq reads mapped to the IPO323 reference genome were analyzed based on transcript-trained, high-quality gene models (82). Read counts were summarized with QTLtools --quan mode. Only reads with a minimum Phred mapping quality >10 were kept for further analyses. Reads were normalized using the --rpkm (reads per kilobase of transcript per million reads mapped) from QTLtools.

### Association mapping analyses for expression variation

SNPs used for the association mapping were retrieved from a variant calling procedure performed with raw sequencing reads checked by FastQC (version 0.11.5) (83) and trimmed with Trimmomatic (75) to remove adapter sequences and low-quality reads with parameters ILLUMINACLIP: TruSeq3-PE.fa:2:30:10 LEADING:3 TRAILING:3 SLIDING WINDOW:4:15 MINLEN:36. Trimmed sequences were aligned to the *Z. tritici* IPO323 reference genome (see above) using bowtie2 version 2.3.4.3 (84) with the option --very-sensitive-local. Variant calling was performed using Haplotypecaller integrated into the Genome Analysis Toolkit (GATK) v. 4.0.11.0 (85). We retained SNPs with QUAL >1000, AN >20, QD >5.0, MQ >20.0, ReadPosRankSum_lower = 2.0, ReadPosRankSum_upper = 2.0, MQRankSum_lower = 2.0, MQRankSum_upper = 2.0, BaseQRankSum_lower = 2.0, BaseQRankSum_upper = 2. Variants passing quality filtration were further filtered to remove multiallelic SNPs using the *bcftools* (version 1.9) --norm option. Variants were filtered to keep only variants genotyped in at least 90% of the individuals and common variants ≥ 5% using the *VCFtools* --max-missing and *bcftools* -q 0.05 minor option (86, 87). Expression quantitative trait loci (eQTL) for variation in locus-specific TE expression were searched using QTLtools (version 1.1) (81) in the *cis* --permutation mode with 1,000 permutations and 5 kb *cis* windows surrounding each TE locus. The permutation *P*-values were false discovery rate (FDR) corrected to identify the top eQTL (5% threshold). We also analyzed gene expression variation associated with TIPs. For this, only genes within 5 kb of each TIP were tested for associations with gene expression levels. Fold change in gene expression was calculated from the ratio of mean gene expression between isolates with TE insertion and isolates without TE insertion. A Wilcoxon test was performed to assess the significance of the association.

### Phenotype-genotype association mapping for TIPs

We analyzed genome-wide TIPs for associations with phenotypic trait variation (i.e., TIP-GWAS). To assess variation in virulence phenotype variation, we performed an infection assay on the Swiss winter wheat cultivar Claro grown in a growth chamber (72). We used diluted (2  ×  10^5^ spores/mL in 15  mL of sterile water containing 0.1% TWEEN2) 8-day-old YSB-grown *Z. tritici* spore suspension to infect the 3-week-old wheat plant. After spray inoculation, the plants were kept at 100% humidity for 21 days. Leaf lesions were assessed using ImageJ. The ratio of the total lesion area and total leaf area was calculated to obtain the percent leaf area covered by lesions (PLACL) (88).

The metabolome composition of the pathogen population was assayed previously using untargeted metabolite profiling based on UPLC-HRMS (52). *Z. tritici* isolates grown in YSB for 8 days were filtered through cheesecloth to remove hyphae and washed in milli-Q water to remove media traces. The spores were suspended and lyophilized to extract metabolites in 1 mL of HPLC-grade methanol. The extract was centrifuged at 15,000 rpm for 5 min to pellet down debris and this last step was repeated until a clear supernatant was recovered. Untargeted metabolite profiling was carried out by UHPLC-HRMS using an Acquity UPLC coupled to a Synapt G2 QTOF mass spectrometer (Waters, Inc.). Formic acid (0.05%) in water as mobile phase A and formic acid (0.05%) in acetonitrile as mobile phase B with a gradient of 0–100% B in 10 min, holding at 100% B for 2.0 min, re-equilibration at 0% B for 3.0 min was used. Samples were analyzed using mass spectrometric parameters of 50–1,200 Da, 0.2 s scan time, 120°C source temperature, 2.5 kV capillary voltage, 25V cone voltage, 900 L/h desolvation gas flow, and 400°C, 20 L/h cone gas flow, and 4 eV collision energy (low energy acquisition function) or 15–50 eV collision energy (high energy acquisition function). Recordings were made using Masslynx XS v.4.1 (Waters Inc.). Detecting markers with Markerlynx XS was performed with the following parameters: initial and final retention times 1.5 and 10 min, mass range 85–1200 Da, mass window 0.02 Da, retention time window 0.08 min, intensity threshold 500 counts, automatic peak width calculations, de-isotoping applied. We used untransformed relative abundance values for each peak from the metabolome analysis for metabolome variation association mapping.

TIPs with a minor allele frequency of >5% (for either the presence or absence of the TE at the locus) were used for association mapping with phenotypic trait variation based on mixed linear models and performed likelihood ratio tests (--lmm 2) with the GEMMA version 0.98.3) (89). The standardized relatedness matrix calculated from individual genotypes was used to correct for uneven relatedness in association mapping. Association *P-*values were considered significant using Bonferroni (90) multiple comparison corrections. The Bonferroni threshold was calculated by dividing the nominal threshold of α = 0.05 by the total number of TIPs used in our GWAS (*n* = 192). In addition, we considered a 5% false discovery rate (FDR) threshold using the *p.adjust* function in the R package *stat* (91). Linkage disequilibrium (*r*^2^) between TIPs and SNPs in a 5 kb upstream and downstream distance from the TIP locus were calculated using the “–hap-r2” in VCFtools v. 0.1.15 (86). Linkage disequilibrium analyses between TIP alleles (presence/absence) and calculated the *r^2^* for all the SNPs in the window to the TIP locus polymorphism. We also calculated *r*^2^ for all pairwise SNP combinations in the same windows.

## Data Availability

All RNA-seq data sets are available from the NCBI Sequence Read Archive BioProject PRJNA596434. Supplementary tables provide all TE insertion, expression, metabolome, and association mapping data and are accessible on Zenodo (https://doi.org/10.5281/zenodo.10020096). [data set] Leen Nanchira Abraham, Croll Daniel; 2022; PRJNA650267 : Population-level transcriptome of *Zymoseptoria tritici*; NCBI Sequence Read Archive.

## SUPPLEMENTAL MATERIAL

Supplemental Figures mbio.02840-23-s0001.pdf Figures S1-S7.

ASM does not own the copyrights to Supplemental Material that may be linked to, or accessed through, an article. The authors have granted ASM a non-exclusive, world-wide license to publish the Supplemental Material files. Please contact the corresponding author directly for reuse.
