# Supplementary material for: Population-level transposable element expression dynamics influence trait evolution in a fungal crop pathogen
Source: mBio. 2024 Feb 13;15(3):e02840-23. doi: 10.1128/mbio.02840-23 (PMC10936205; doi:10.1128/mbio.02840-23)
Supplement: Supplemental Figures — Figures S1-S7. [file mbio.02840-23-s0001.pdf]

Supplementary Information

Population-level transposable element expression dynamics influence trait evolution in a fungal crop pathogen

Leen Nanchira Abraham, Ursula Oggenfuss, Daniel Croll

Supplementary Figures S1-S7

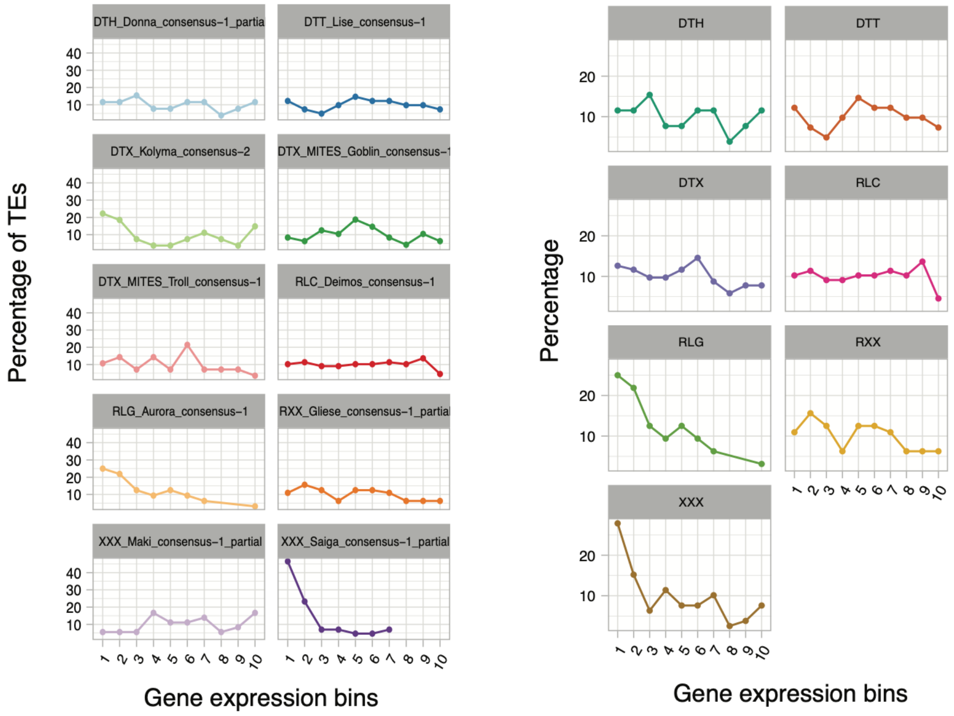

Supplementary Figure S1: Genome-wide TE insertion profile neighboring to the genes. Genes are categorized into bin by increasing order of gene expression (RPKM). Individual TE loci are grouped by TE superfamilies.

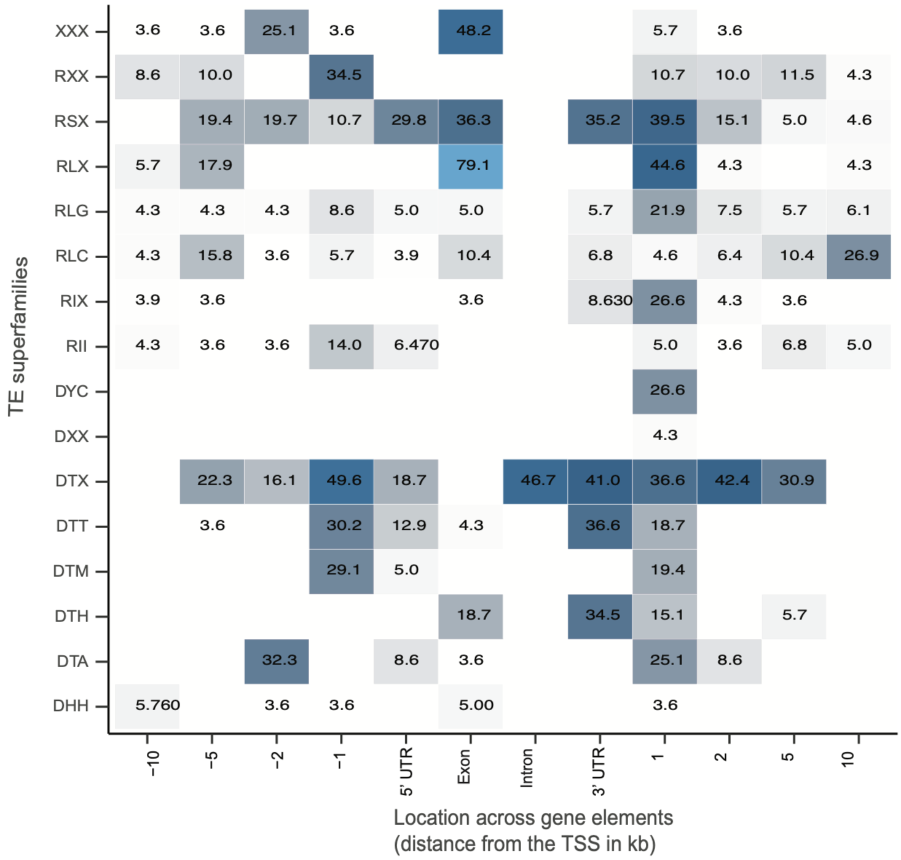

Supplementary Figure S2: Frequency of different TE superfamilies in the population within the gene elements. The numbers in the coloured boxes represent the percent of isolates in the population that carrying TE insertion in the corresponding gene elements. The colour gradient from grey to dark blue correspond with the increasing order of TE insertions.

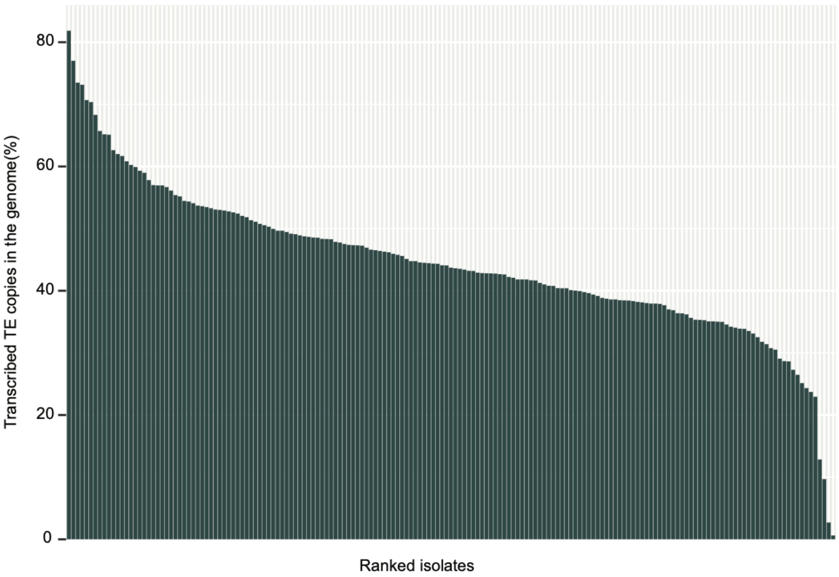

Supplementary Figure S3: Percent of transcribed TE copies in the genome of isolates in the population.

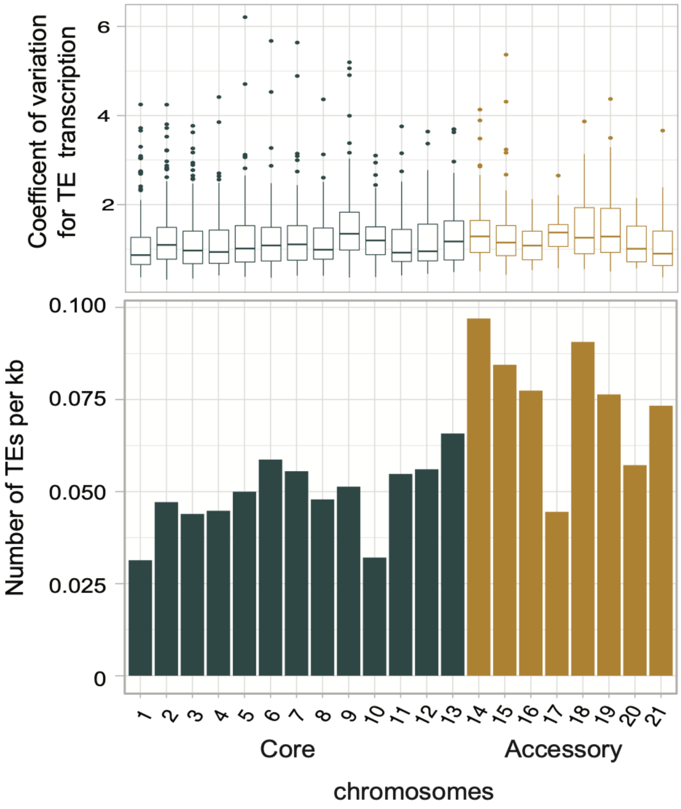

Supplementary Figure S4: Population-level transcriptional variation of individual TE copies binned by chromosome. Distribution of TE copies across chromosomes.

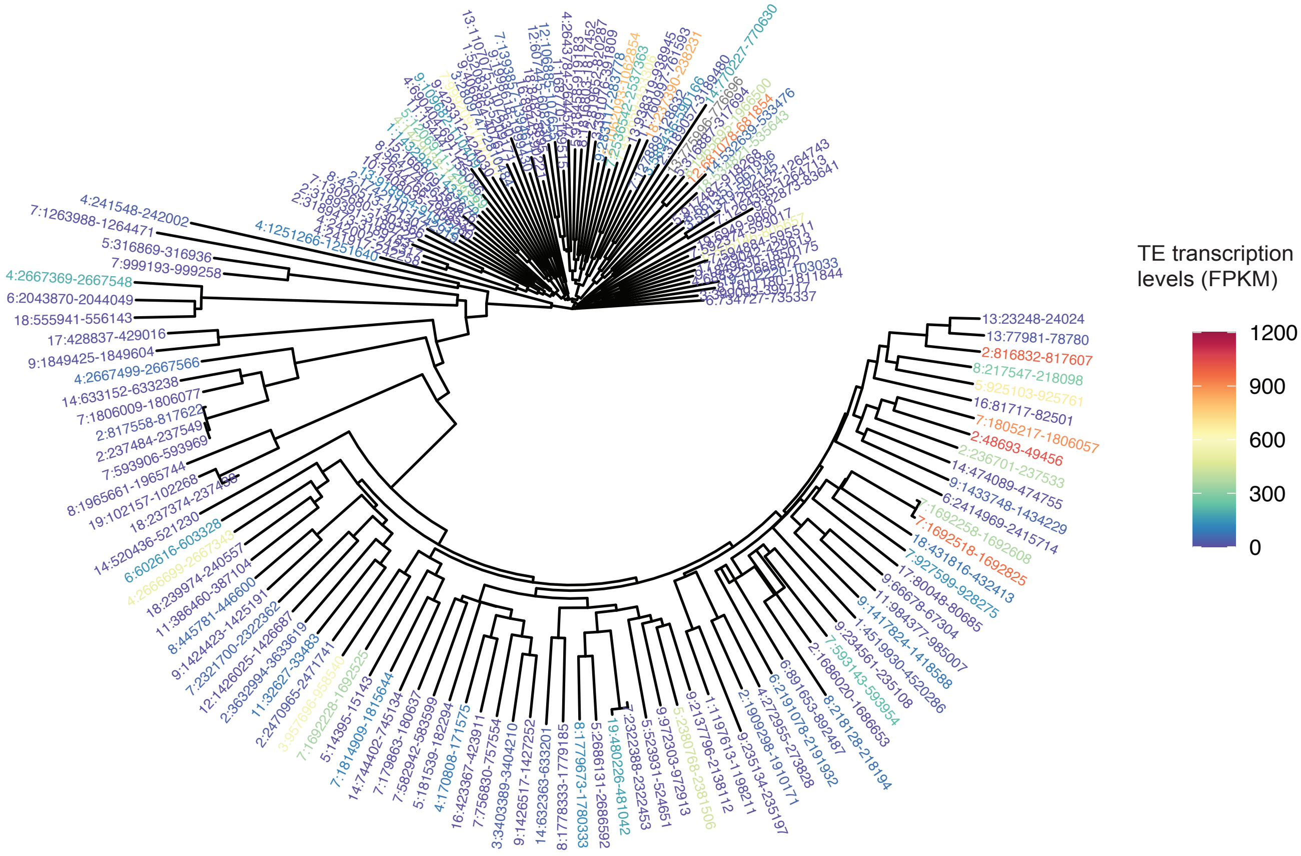

Supplementary Figure S5: Neighbor-joining tree of all copies of the RLX_Lard_Gridr family discovered in the IPO323 reference genome. Colors highlight transcription levels for each copy.

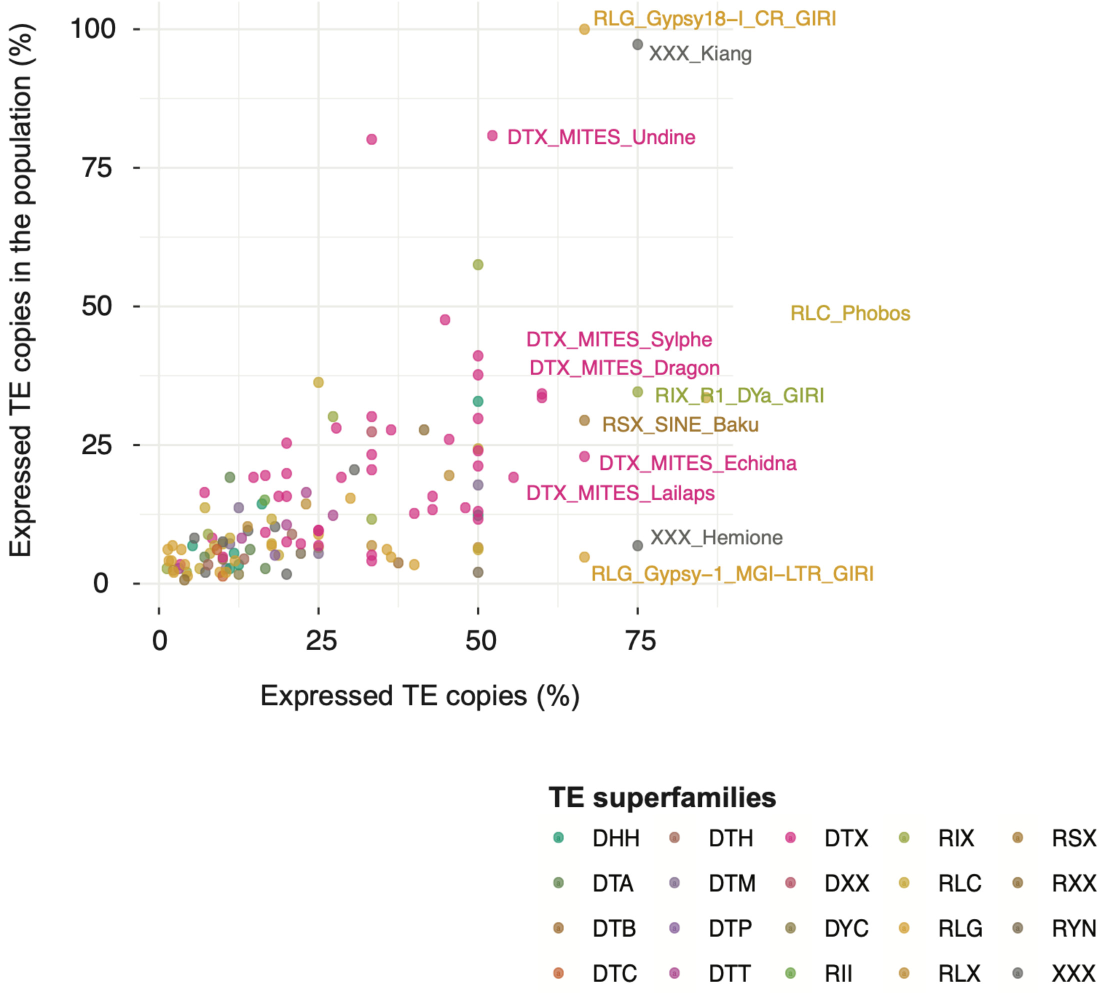

Supplementary Figure S6: Percent of expressed TE copies in the reference genome isolate IPO323 and percent of TE copies expressed across the population. Each dot represents a TE family and the percentages refer to different copies of the same family. Colors indicate superfamily groups.

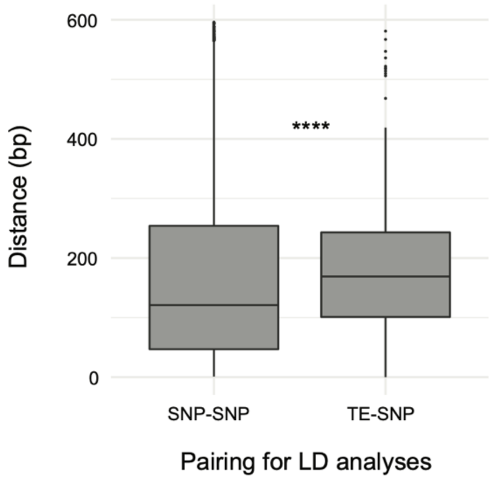

Supplementary Figure S7: Distances between pairs of SNPs, and pairs of TIPs with SNPs in the 5 kb upstream and down steam from a focal TIP position.

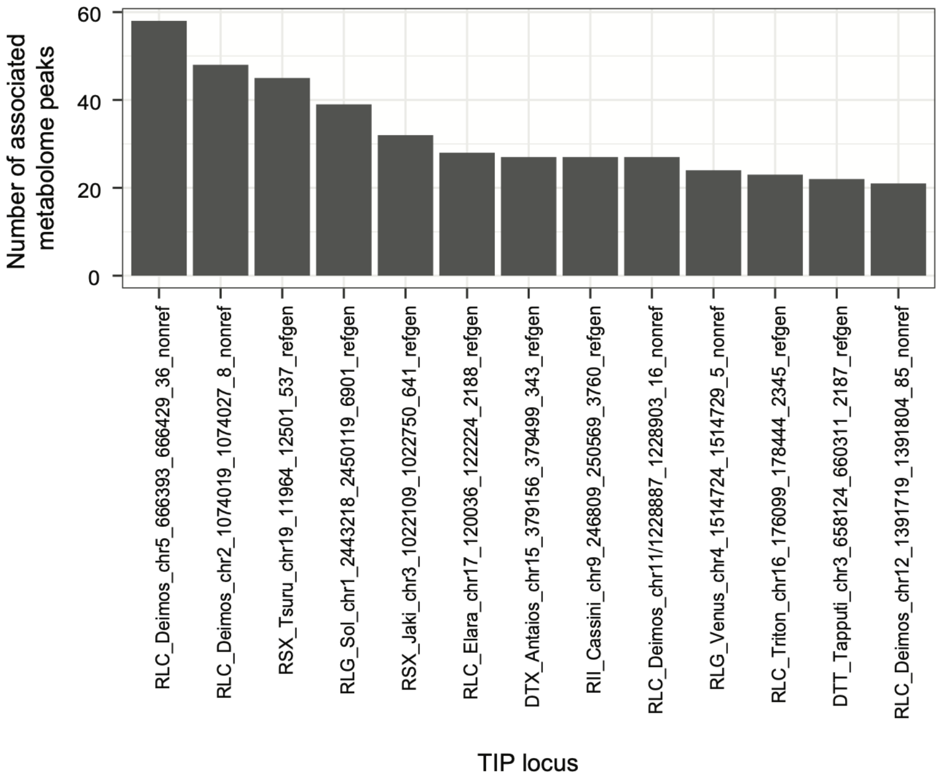

Supplementary Figure S8: TIPs significantly associated with variation in ≥20 metabolite peak profiles.
